# Genetic overlap between multi-site chronic pain and cognition: a large-scale genome-wide cross-trait analysis

**DOI:** 10.3389/fnins.2025.1466278

**Published:** 2025-03-14

**Authors:** Yanjing Chen, Jiankai Deng, Zhiyi Zhang, Chenlin Wang, Xuegao Yu

**Affiliations:** ^1^Department of Radiology, Second Xiangya Hospital, Central South University, Changsha, Hunan, China; ^2^Department of Laboratory Medicine, The First Affiliated Hospital, Sun Yat-sen University, Guangzhou, China; ^3^Department of Massage, Quanzhou Orthopedic-Traumatological Hospital, Quanzhou, Fujian, China; ^4^Fujian University of Traditional Chinese Medicine, Fuzhou, Fujian, China

**Keywords:** pain, cognition, genetic architecture, genetic correlation, GWAS

## Abstract

**Background:**

Different studies have consistently demonstrated a positive correlation between chronic pain and cognitive changes. This study aimed to explore the genetic factors underlying the relationship between chronic pain and cognitive traits, and to investigate whether an inherent causal connection exists between them.

**Method:**

The genetic contributions of chronic multi-site pain and eight cognitive traits were investigated based on Genome-wide association studies (GWAS) data. Linkage disequilibrium score regression (LDSC) was employed to assess the genetic correlations between each pair of traits. The shared genetic components of these traits were investigated by identifying single nucleotide polymorphisms (SNPs) with pleiotropic effects using the Cross Phenotype Association (CPASSOC) method. Furthermore, enrichment analysis and transcriptome-wide association studies (TWAS) were performed to characterize the significant associations between genetic traits. The latent causal variable model (LCV) was employed to explore the potential causal relationship between both traits.

**Results:**

A significant negative genetic correlation was found between chronic pain and several cognitive functions, particularly intelligence (rg = −0. 11, *p* = 7.77 × 10^−64^). CPASSOC identified 150 pleiotropic loci. A co-localization analysis was conducted, which identified 20 loci exhibiting pleiotropic effects at the same genomic position. The LCV analysis indicated no causal relationship between both traits.

**Conclusion:**

The present work contributed to an enhanced understanding of the complex genetic interplay between cognitive function and chronic pain.

## 1 Introduction

The prevalence of chronic pain has been progressively increasing, leading to a growing societal burden on a global scale, especially among older adults. It has been reported that in the United States, more than 50 million adults (20.5%) suffer from pain most days (Woolf, 2004). Chronic pain is characterized as persistent pain lasting for a minimum duration of 3 months, affecting one or more systems of the body (Pitcher et al., 2019). Previous studies have observed that 20% of chronic pain patients, specifically those without a prior diagnosis of neurological disorders, may encounter cognitive impairment, which can significantly impact their social and daily activities (van Hecke et al., 2013).

Individuals with chronic pain affecting multiple body regions are reported to have a twofold increased risk of developing dementia compared to those with pain localized to a single body region. This increased risk is not influenced by factors such as medication use, economic status, or coexisting medical conditions (Picavet et al., 2002). The brain regions responsible for processing pain, including the thalamus and somatosensory cortex, and those involved in cognitive functions, such as the prefrontal cortex and hippocampus, develop rapidly during infancy and early childhood (Addabbo et al., 2021). Early experiences of pain or pain-related stimuli may influence children’s cognitive and emotional development (Goubert et al., 2005). Pain perception mediates its emotional impact at the neuroanatomical and neurobiological levels, including alterations in the limbic system (Tracey, 2010). Conversely, improved cognitive ability can augment positive emotional experiences, influencing an individual’s ability to cope with pain (Khera and Rangasamy, 2021). Furthermore, the utilization of analgesics has been found to have a detrimental impact on cognitive function (Johansson et al., 1998). Hence, additional confounding variables introduce ambiguity to the observed association between chronic pain and cognitive functioning in epidemiological research, requiring further examination to establish a causal link between the two.

Genes play a crucial role in the regulation (Einarsdottir et al., 2004) and perception of pain (Testa et al., 2021), while cognitive performance is also a heritable trait (Procopio et al., 2022). Assuming the etiologies of chronic pain and cognitive function overlap, there may be an underlying genetic basis for both traits. The study conducted a Genome-wide association studies (GWAS) analysis of chronic pain and used genetic correlation methods to identify a negative association between chronic pain and cognition (OR [95% confidence interval] = 0.69 [0.53–0.89], *p* = 0.043) (Guo et al., 2023). A study using a murine migraine model highlighted the involvement of the P2X7R/NLRP3 signaling pathway in neuroinflammation, glioblast development, and cognitive impairment-related neuronal loss (Wang et al., 2022) Although some studies suggest a correlation between genetic factors, the precise genetic structure or causal relationship behind the association between these traits has yet to be thoroughly investigated, and further research is needed to substantiate these findings.

To clarify the relationship between chronic pain and 8 kinds of cognitive performance, the data from the largest available GWAS was analyzed for these traits to reveal the shared genetic architecture. The present study identified possible pharmacological targets that may have the capacity to mitigate cognitive impairment in persons suffering from chronic pain over an extended period.

## 2 Method

### 2.1 The source of GWAS

**Figure 1:**
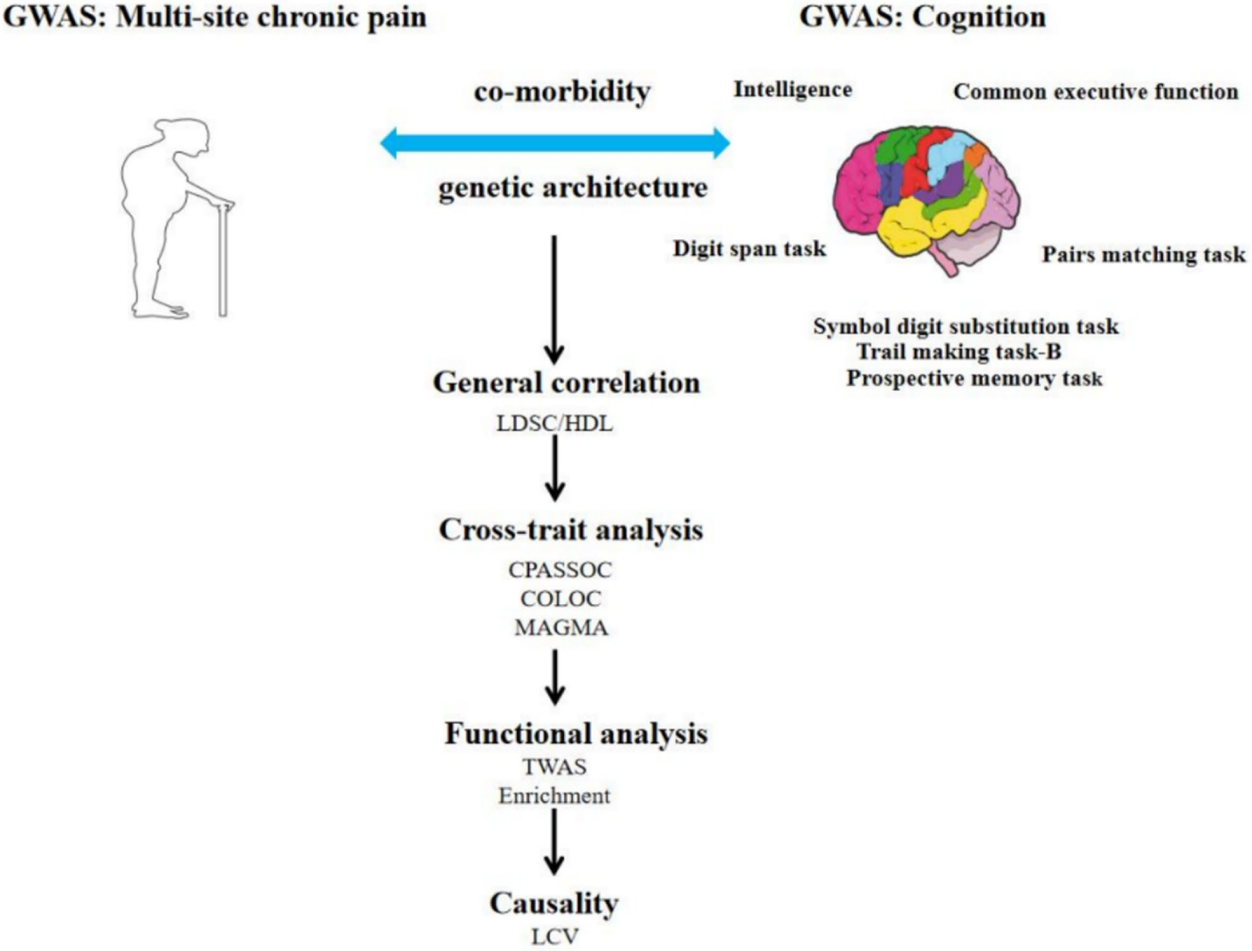
Overview of research of shared genetic architecture between multi-site chronic pain and cognition.

To evaluate the relationship between cognitive functions and chronic pain, the cognitive abilities were categorized into prospective memory task, pairs matching task, trail-making test part B, symbol digit substitution task, digit span task, reaction time, intelligence, and common executive function (Table 1). We obtained GWAS summary statistics from two recent publications for cognitive functions (Hatoum et al., 2023) and multi-site chronic pain (Johnston et al., 2019). The data was standardized by retaining only single nucleotide polymorphisms (SNPs) from the 1,000 Genomes European population (Auton et al., 2015), removing SNPs without rsID or with duplicate rsID, performing genotype imputation to fill in missing data using reference datasets like the 1,000 Genomes Project, and referencing the genomic positions to the hg19 reference genome. The flowchart can be seen in Figure 1.

**Table 1:** The detailed information of the source of GWAS of cognition and multi-site chronic pain.

Diseases	Abbreviations	PMID	Year	N_total	Ancestry
Multi-site chronic pain	MCP	31194737	2019	387,649	EUR
Common executive function	CE	36150907	2022	427,037	EUR
Intelligence	IG	36150907	2022	216,381	EUR
Reaction time	RT	36150907	2022	432,297	EUR
Prospective memory task	MT	36150907	2022	162,335	EUR
Pairs matching task	PMT	36150907	2022	81,701	EUR
Trail making task-B	TMT	36150907	2022	93,024	EUR
Symbol digit substitution task	SDST	36150907	2022	84,125	EUR
Digit span task	DST	36150907	2022	81,701	EUR

### 2.2 Genetic correlation analysis

The pairwise genetic correlation analysis was conducted using the linkage disequilibrium score regression (LDSC) (Bulik-Sullivan et al., 2015; Bulik-Sullivan et al., 2015). LDSC measures the average extent to which genetic effects, represented by heritability (h^2^) and genetic correlation (rg), are shared across the entire genome for two traits. This analysis is not affected by environmental confounders. A Bonferroni-corrected *p*-value threshold of *p*: 0.05/8 = 6.25 × 10^−3^ was used to represent statistical significance. The pre-computed linkage disequilibrium (LD) scores were obtained from ~1.2 million common SNPs in European ancestry represented in the HapMap3 reference panel. High-definition likelihood inference (HDL) is an alternative approach for analyzing genetic association (Ning et al., 2020). Unlike LDSC, HDL leverages individual-level summary statistics from GWAS data and utilizes a more comprehensive LD matrix to accurately model the relationship between test statistics (such as effect sizes or *p*-values) and LD structure, enabling finer resolution in identifying genetic correlations. The R program HDL-v1.4.0^1^ was utilized to perform HDL, using the reference panel of 1,029,876 correctly imputed HapMap3 SNPs^2^.

### 2.3 Cross-trait analysis

A cross-trait meta-analysis (CPASSOC) (Zhu et al., 2015) combines summary data from several correlated traits obtained from GWAS to identify genetic variants associated with at least one of these traits. CPASSOC is a statistical method for cross-trait meta-analysis that integrates summary-level data from multiple correlated GWAS. This not only improves the statistic efficacy but also increases the effective sample size. Significant pleiotropic SNPs showed a genome-wide significance of *p*CPASSOC = 5 × 10^−8^ in paired traits and a significance of *p*single trait = 1 × 10^−3^ in a single trait. The present work presented the hypothesis that the meta-analysis significance, using a threshold of *p* < 5 × 10^−8^, in addition to trait-specific significance ranging from 5 × 10^−8^ to 1 × 10^−3^, suggests the presence of novel shared loci between the two traits.

Given that the preceding research relied on a meta-based approach, the outcomes produced may encompass risk genomic regions that are exclusively linked to a single trait. Hence, additional inquiry is important to ascertain if two traits are caused by the same set of genetic variants or by distinct variants that are in close proximity. Furthermore, a colocalization analysis (COLOC) (Wallace, 2020) was carried out using the Bayesian algorithm to generate posterior probabilities for five mutually exclusive hypotheses about the sharing of causal variants in a genomic region, namely H0 (no association), H1 or H2 (association with one trait only), H3 (association with both traits, two distinct SNPs), and H4 (association with both traits, one shared SNP). At each shared locus, the summary data was retrieved for the variations located within 5 Mb of the index SNP, and the posterior probabilities for H4 (PPH4) and H3 (PPH3) were computed. The PPH4 greater than 70% was considered a significant genetic variation associated with both traits.

To aid in addressing challenges in interpreting SNP effects, such as limited functional insights, pleiotropy, and complex LD structures, we used Functional mapping and annotation (FUMA) to annotate SNPs, particularly those located in non-coding regions, by mapping them to genes and determining their functional relevance. FUMA is a functional annotation platform designed to identify independent genomic loci, providing detailed information on SNPs, including functional categories, CADD scores, RegulomeDB scores, and chromatin states. Specifically, FUMA was utilized to map genes within 500 kb of each CPASSOC-identified candidate SNP and prioritize them based on LD with genome-wide significant SNPs at the adjusted r^2^ threshold. Furthermore, the Multi-marker Analysis of GenoMic Annotation (MAGMA) (de Leeuw et al., 2015) was used, which is a recently developed tool for gene and gene-set analysis. This tool utilizes a regression structure that effectively incorporates the LD between SNPs, surpassing the capabilities of other existing approaches. It evaluates gene-level associations by aggregating SNP data while accounting for LD and incorporating functional annotations to improve biological relevance, and it also computes the joint association of multiple gene sets with phenotypes During the analysis, genes within 500 kb of each candidate SNP were mapped and prioritized if they were in LD with the genome-wide significant SNPs at the adjusted *r*^2^ threshold. The intersection of genes obtained from the MAGMA algorithm and the genes annotated by CPASSOC that exhibited greater robustness in terms of pleiotropy after adjustments were considered for analysis.

### 2.4 Functional analysis

Functional analysis of genes investigates the impact of genes and their variants on biological functions, expression, and pathways to reveal their mechanisms in phenotypic outcomes. A transcriptome-wide association study (TWAS) (Gusev et al., 2016) can identify genes whose expression is significantly associated with complex traits. The TWAS algorithm employs a limited number of reference individuals to assess gene expression and extrapolate the expression patterns to a wider group of phenotyped individuals based on their SNP genotypes. The reference used here is sourced from Functional Summary-based Imputation (FUSION)^3^, a method that constructs predictive models to estimate the genetic contribution to functional or molecular phenotypes. In this study, we applied predictive models derived from brain and whole blood tissues, as these tissues are most relevant to the phenotype under investigation. Specifically, brain tissue models provide insights into neurological aspects of the trait, while whole blood models capture systemic and immune-related genetic effects. To further explore the biological functions of these genes, the enrichment of the shared genes was assessed and detected by MAGMA, while the annotation results of CPASSOC in Gene Ontology (GO) biological processes (Gene Ontology Consortium, 2015) and Kyoto Encyclopedia of Genes and Genomes (KEGG) pathways were also assessed (Ogata et al., 1999). The GO analysis provides hierarchical annotations of the genes in terms of their biological processes (BP), molecular functions (CF), and cellular components (CC). The Rstudio “Cluster package” was utilized for this enrichment analysis. Additionally, MAGMA was used for gene-tissue enrichment analysis, utilizing reference templates from the GTX-8 database, which comprises a total of 54 tissue templates. These templates were specifically selected to identify tissues that are significantly associated with cognitive function, providing insights into the biological relevance of the identified genes in relation to the phenotype.

### 2.5 Causality

To identify if the correlation contains a causal component, the latent causal variable (LCV) model (Ning et al., 2020) was used, which is based on the hypothesis that a latent variable mediates the genetic correlation between two traits. Compared to Mendelian randomization (MR), the present study demonstrates a more robust assessment of the causal association between traits, as it mitigates the potential confounding effect arising from the genetic correlation between the two traits. The genetic causality proportion (GCP) can indicate the direction of causality. A value closer to 1 indicates a stronger causal effect of trait 1 on trait 2. A negative value denotes the proportion to which the genetic component responsible for trait 2 influences trait 1. Rho represents the genetic correlation between the two traits, estimated using LD score regression.

## 3 Results

### 3.1 Genetic correlation analysis

**Figure 2:**
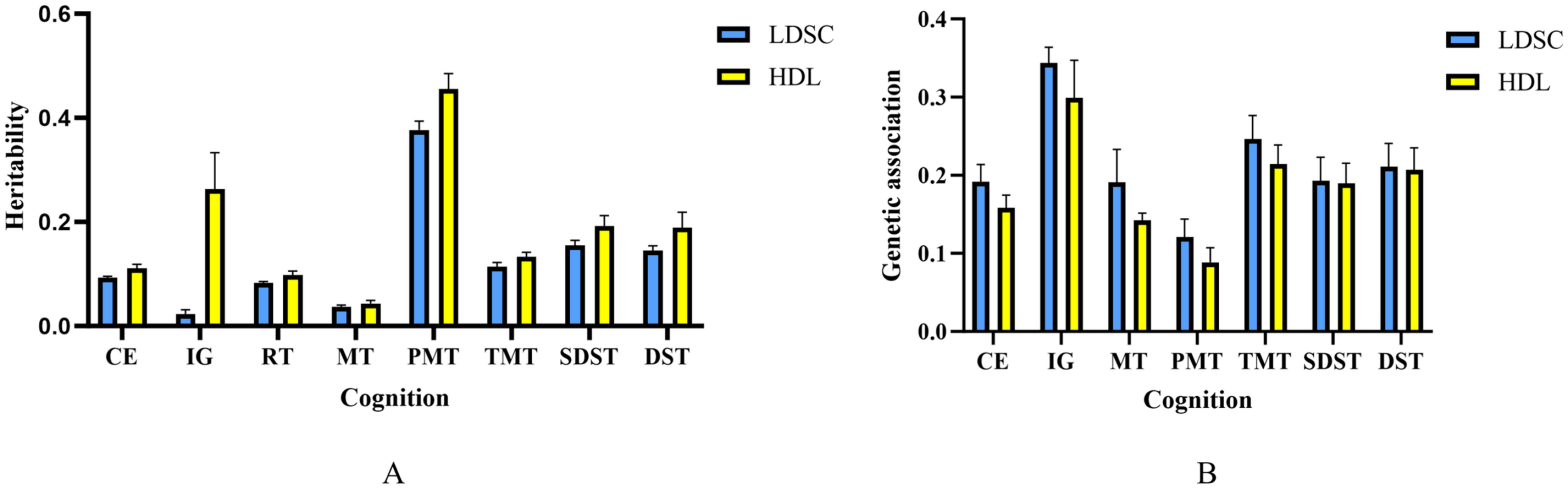
Cognitive trait heritabilities **(A)** and genetic correlations with chronic pain **(B)** error bars represent SE.

The heritability of chronic pain was 0.077, detected by LDSC. Except for the genetic correlation between RT and chronic pain (rg = 0.0307, *p* = 3.07 × 10^−2^), the genetic association between most paired traits was significantly negative like the paired trait of chronic pain and intelligence and the paired trait of chronic pain and CE (rg = −0. 19; *p* = 1.10 × 10^−18^). HDL analysis indicated that the heritability of the pairs matching task was the highest (*h*^2^ = 0.4554), which is roughly consistent with the LDSC results (Figure 2). The results for other traits are presented in Supplementary Table S1, including heritability and the standard error (SE) of genetic correlations.

### 3.2 Cross-trait analysis

A total of 150 pleiotropic loci were shared between chronic pain and cognition (Supplementary Table S2). Among these loci, 56 were identified for the CP-IG trait pair, 68 for CP-CE, and 26 for chronic pain and other cognitive functions. Notably, the corresponding topSNP in the most significant locus shared between chronic pain and intelligence was identified as rs13135092 (*p*chronic pain = 7.20 × 10^−15^, *p*intelligence = 9.10 × 10^−22^; *p*meta-analysis = 2.78 × 10^−25^), mapping to two genes: *BANK1* and *SLC39A8*. The SNP rs13135092 was also the top shared variant between chronic pain and common executive function (*p*chronic pain = 5.50 × 10^−8^; *p*common executive function = 7.20 × 10^−15^; *p*meta-analysis = 2.79 × 10^−18^). The meta-analysis identified 83 novel pleiotropic loci jointly associated with chronic pain and cognitive function, defined as *p* < 5 × 10–8 and trait-specific significance ranging between 5 × 10^−8^ and 1 × 10^−3^ (Supplementary Table S3). Among these, 26 loci were specifically shared between chronic pain and intelligence, with a majority of them showing opposite effect directions. Additionally, 47 loci were shared between chronic pain and common executive function, and 10 loci were shared between chronic pain and other cognitive functions. Further colocalization analysis identified 57 statistically significant genes (Supplementary Table S4). Including 20 loci classified as potential pleiotropic loci (PPH4 > 0.7). Each locus had a corresponding topSNP, identified as a candidate-shared causal variant by CPASSOC (*p*meta-analysis <5 × 10^−8^; *p*trait-specific <0.001) (Table 2). Notably, one pleiotropic locus associated with four trait pairs was colocalized with the corresponding paired traits, including CP-IG (PPH4 = 0.99). The same shared causal variant, rs13135092, was also identified. COLOC further identified the gene NCAM1 at shared loci for CP-SDST, CP-PMT, and CP-IG trait pairs, consistent with CPASSOC results.

**Table 2:** Significant results from colocalization analysis for each pleiotropic locus identified from CPASSOC.

TopSNP	Chromosome	GenomicLocus	A1	A2	Chronic pian				Beta	Cognition *Z*	*p*-value	*P*CPASSOC	PPH3	PPH4
					Beta	Z	*p*-value	Trait						
rs13135092	4	63	G	A	0.03	7.39	7.20E-15	IG	−0.04	−9.59	9. 10E-22	2.78E-25	0.001	0.999
rs7105462	1	138	G	A	0.01	5.34	1.90E-07	IG	−0.01	−4.43	9.50E-06	2.78E-10	0.215	0.739
rs13135092	4	9	G	A	0.03	7.39	7.20E-15	TMT	−0.05	−5.23	1.70E-07	1.24E-15	0.002	0.998
rs13135092	4	55	G	A	0.03	7.39	7.20E-15	CE	−0.01	−5.43	5.50E-08	2.79E-18	0.001	0.999
rs13135092	4	9	G	A	0.03	7.39	7.20E-15	MT	−0.01	−5.78	7.40E-09	1.43E-17	0.001	0.999
rs7105462	11	95	G	A	0.01	5.34	1.90E-07	PMT	0.01	4.73	2.30E-06	1.00E-09	0.120	0.880
rs7105462	11	35	G	A	0.01	5.34	1.90E-07	SDST	−0.02	−4.67	3. 10E-06	4.59E-09	0.065	0.907

At the genetic level, the MAGMA identified 2,157 potential pleiotropic genes that overlapped with the physical annotation (Figure 2, Supplementary Table S5). After Bonferroni correction, 1,679 of these genes remained significant (Supplementary Table S6). Among them, 119 genes were shared between SDST and chronic pain, with apolipoprotein C1 (*APOC1*) being the most significant gene (*p* = 2.30 × 10^−14^). This gene was also shared between PMT, TMT, CE, and chronic pain. Additionally, 324 were shared between PMT and chronic pain, 108 between TMT and chronic pain, 406 between CE and chronic pain, and 531 between IG and chronic pain. The most significant gene shared between CP-IG was DCC (*p* = 2.33 × 10^−27^), which was also shared with other functional traits and chronic pain. Furthermore, RNA binding fox-1 homolog 1 (*RBFOX1*) was the most significant gene shared between CE and chronic pain (*p* = 1.92 × 10^−17^).

### 3.3 Functional analysis

A total of 1,036 genes remained significant after correction in the TWAS analysis (Supplementary Table S7). Among them, 400 genes showed significant expression in multiple tissues (Supplementary Table S8). The highest number of significantly expressed genes was observed in both blood and the cerebral cortex, with 48 genes identified in each tissue. In the cerebral cortex, the most significantly expressed gene for CP was ring finger protein 123 (*RNF123*) (*p* = 6.44 × 10^−30^), with a corresponding IG expression of *p* = 5 × 10^−10^. Conversely, the most significantly expressed gene for IG was glutathione peroxidase 1 (*GPX1*) (*p* = 5.16 × 10^−15^). Additionally, 11 genes were significantly expressed in the pair trait CP-CE, representing the second-highest number of genes, while 10 genes were identified in CP-PMT/CP-TMT. Furthermore, 49 genes with significant expression in the brain’s nucleus accumbens (basal ganglia) were also identified.

**Figure 3:**
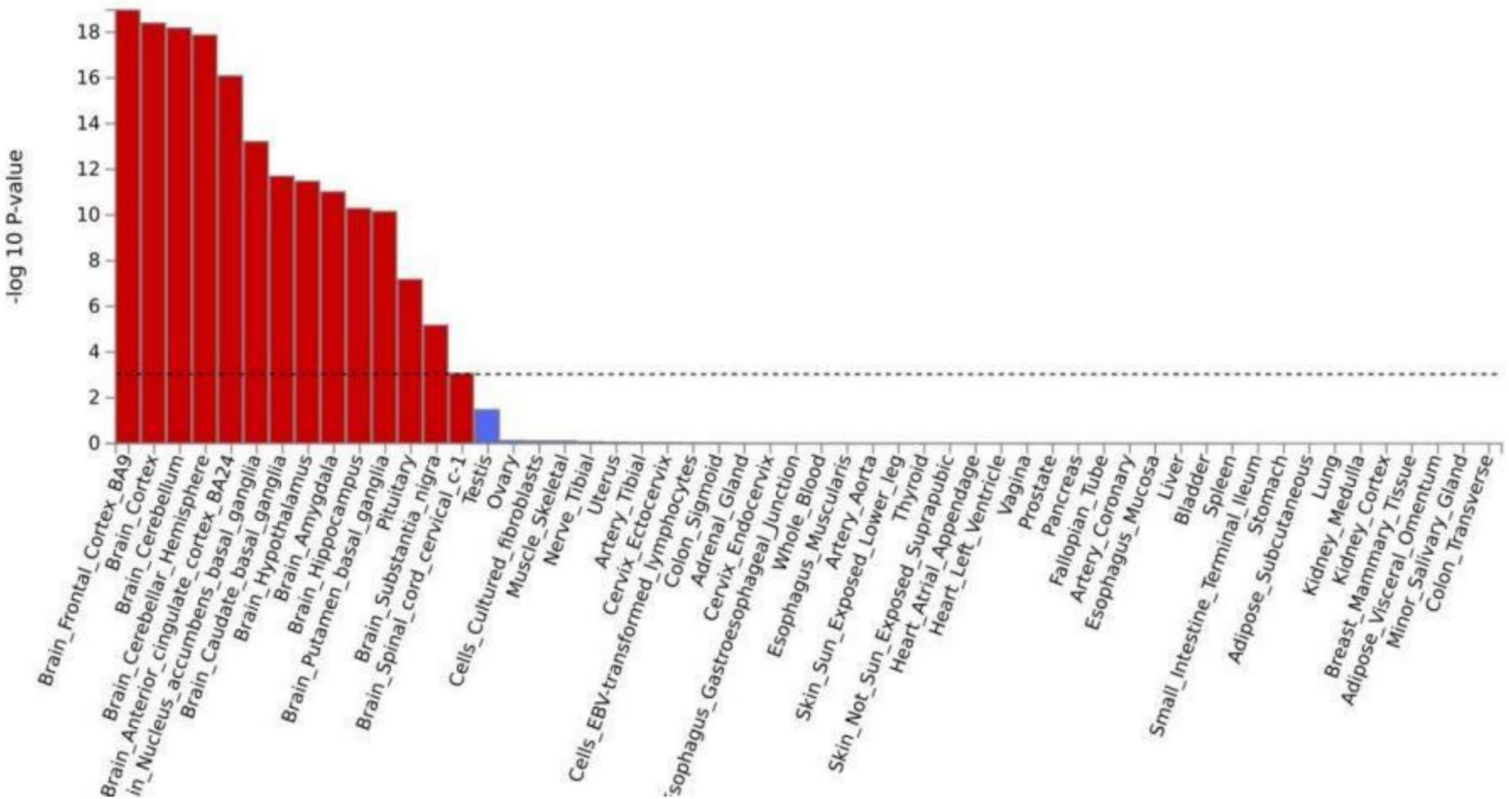
Tissue enrichment in GTEx version 8 for the results of multi trait of cognition and chronic pain.

Tissue analysis revealed that the genes associated with both cognition and chronic pain were predominantly enriched in brain tissues. After applying the false discovery rate (FDR) correction, significant enrichment was observed in 13 brain tissues (Supplementary Table S9). Notably, shared loci between IG/SDST and chronic pain were most significantly enriched in the frontal cortex (*p*IG = 5.03 × 10^−24^, *p*SDST = 1.09 × 10^−9^). Similarly, the shared loci between MT/DST and chronic pain demonstrated prominent enrichment in the cerebellum of the brain (*p*IG = 5.03 × 10^−24^, *p*SDST = 1.09 × 10^−9^). For TMT and chronic pain, the shared loci were enriched in the cortex (*p*TMT = 2.73 × 10^−9^), while CE and chronic pain exhibited significant enrichment in the cerebellar hemisphere (*p*CE = 2.35 × 10^−17^). Tissue enrichment was also analyzed based on the result of CPASSOC of the 8 paired traits (Supplementary Table S10, Figure 3).

**Figure 4:**
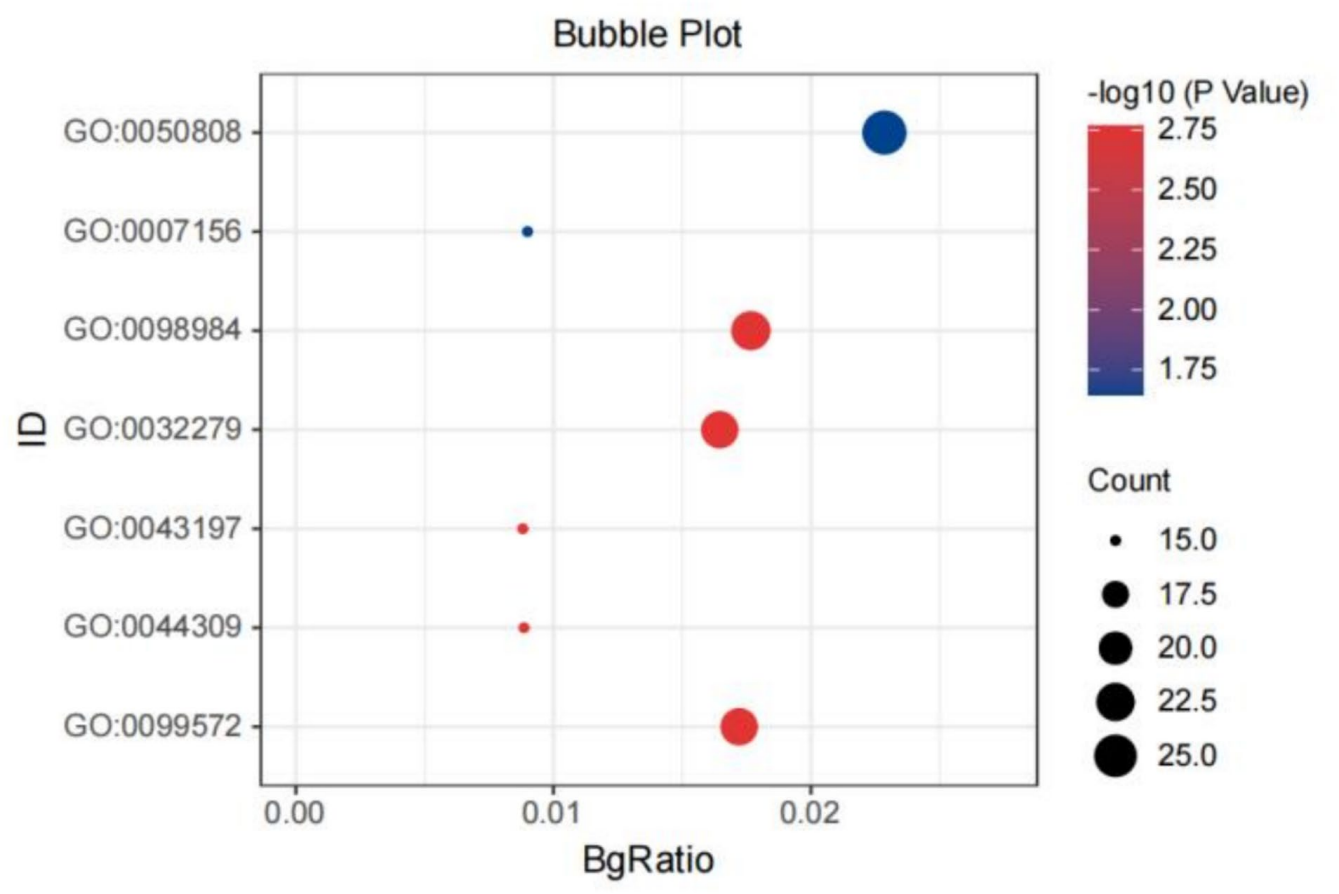
The bubble plot of the GO analysis of the shared genes between IG and chronic pain. GO: 0050808: synapse organization; GO: 007156: homophilic cell adhesion via plasma membrane; adhesion molecules; GO: 0098984: neuron to neuron synapse; GO: 0032279: asymmetric synapse; GO: 0044309: neuron spine, GO: 0099572: postsynaptic specialization.

GO analysis revealed significant enrichment for shared genes between chronic pain and intelligence in synapse organization (*p* = 1.07 × 10^−5^, *p*adjust = 0.023; Figure 4). For shared genes between CE and chronic pain, BP pathways passing the significance threshold included neuron projection development (*p* = 4.04 × 10^−6^, *p*_adjust_ = 0.0129). As well as those related to regulation of synaptic plasticity (*p* = 1.74× 10^−5^, *p*_adjust_ = 0.016) and muscle development (*p* = 1.28 × 10^−5^, *p*_adjust_ = 0.015). CC enrichment for shared genes between CE and chronic pain was primarily in dendritic spines/neuron spines (*p* = 5.48 × 10^−5^, *p*adjust = 7.9 × 10^−3^). The BP of the shared genes between SDST (*p* = 3.42 × 10^−5^, *p*adjust = 0.045) or TMT (*p* = 1.50 × 10^−6^, *p*adjust = 2.71 × 10^−3^) and chronic pain were mainly enriched in the development of skeletal muscles. KEGG pathway analysis showed that shared genes between TMT, SDST, DST, and chronic pain were enriched in the phosphatidylinositol signaling system. Among these, the enrichment of the shared genes between TMT and chronic pain was particularly significant (*p* = 4.74 × 10^−5^, *p*adjust = 6.19 × 10^−3^; Supplementary Tables S11, S12).

### 3.4 Causality

The analysis revealed that when CP was used as the exposure, only PMT (*ρ* = 0.11, SE = 0.03) and TMT (*ρ* = 0.06, SE = 0.02) showed positive genetic correlation values with cognitive traits as outcomes. These positive correlations suggest a potential shared genetic architecture between chronic pain and these cognitive traits. However, the index of causality (*ρ*) was not statistically significant, suggesting that the observed associations do not imply a direct causal relationship (Table 3).

**Table 3:** The result of latent causal variable model for each pair trait.

Trait1	Trait2	Estimated posterior gcp	*p*	Estimated rho
CP	IG	−0.01 (0.05)	0.922	−0.37 (0.03)
CP	PMT	0.32 (0.45)	0.308	0. 11 (0.03)
CP	TMT	0.02 (0.54)	0.744	0.06 (0.02)
CP	DST	0. 18 (0. 18)	0.435	−0.23 (0.04)
CP	CE	0.31 (0.29)	0.384	−0. 19 (0.04)
CP	MT	−0. 13 (0. 18)	0.366	−0. 18 (0.05)
CP	SDST	0.31 (0.29)	0.380	−0. 19 (0.04)

GCP, genetic causality proportion; rho, the genetic correlation.

## 4 Discussion

This study examined the genetic architecture linking pain and eight cognitive traits based on GWAS. In the present investigation, a significant negative genetic correlation between chronic pain and most of the cognitive functions was found. The use of meta-cross-trait analysis led to the identification of 150 pleiotropic loci that exhibited associations with either one or both traits. Notably, 83 of these loci were newly identified by this research. Subsequently, 1,679 pleiotropic genes were identified, and the LCV model was utilized to explore whether causal factors drive the genetic correlation between the traits.

Regardless of whether it is the SNP-level analysis, including CPASSOC and COLOC, or the gene-level analysis using MAGMA. It was found that neural cell adhesion molecule 1 (*NCAM1*) is a genetic factor associated with both chronic pain and various cognitive functions, including intelligence, perceptual-motor tasks, cognitive efficiency, and symbol digit substitution test. *NCAM1* encodes the neural cell adhesion molecule 1, which is a glycoprotein that regulates the growth and branching of axons and dendrites (Piras et al., 2015). The occurrence of peripheral damage has been seen to result in the long-term augmentation of synaptic responses. This phenomenon has been associated with alterations in the plasticity of the cerebral cortex (Zhuo, 2016).

Prior research has suggested that the increased conversion of the synaptic protein *NCAM1* inside the anterior cingulate cortex plays a significant role in the persistent hypersensitivity observed in cases of neural injury (Ko et al., 2018). In addition, *NCAM1* has been confirmed as one of the adhesion molecules expressed by astrocytes (Liddelow and Hoyer, 2016). These molecules might facilitate the interaction between astrocytes and lymphocytes, thereby facilitating the recruitment of immune cells to the central nervous system. According to a cognitive perspective, a study revealed that mice with deficiencies in the three major isoforms of *NCAM1* had learning difficulties (Cremer et al., 1994; Stork et al., 1997). Epidemiological research has also suggested that *NCAM1*, tetratricopeptide repeat domain 12 (*TTC12*), and zinc finger and BTB domain containing 20 (*ZBTB20*) were the three significant genetic loci associated with age-related cognitive decline (Lin et al., 2021). With the increasing incidence of chronic diseases and pain, especially in the middle-aged and elderly population, this may work as an effective drug target to slow down the rate of cognitive decline.

Intellectual decline was the cognitive attribute most significantly linked to chronic pain genetically (Lin et al., 2021). It has been shown that *ERBB3* (epidermal growth factor receptor 3), a member of the epidermal growth factor receptor (EGFR) family, plays a significant role in the pathogenesis of pain (Verma et al., 2020; Mitchell et al., 2020). Pharmacological inhibition of EGFR using small molecules or monoclonal antibodies has demonstrated analgesic effects in individuals with chronic pain ^[30]^. Additionally, research has shown that the absence of ErbB3 receptors in oligodendrocytes leads to altered prefrontal cortex functioning and impaired myelin development in solitary mice, further highlighting the potential role of EGFR-related pathways in pain and associated cognitive processes (Makinodan et al., 2012).

Additionally, the present genetic investigation revealed that the locus with the highest level of significance, which is shared between cognition and pain, corresponds to the genes apolipoprotein E (*APOE*), *APOC1*, and translocase of outer mitochondrial membrane (*TOMM4*). Numerous studies have consistently identified these genes as pivotal contributors to Alzheimer’s disease and mild brain injury. Astrocytes predominantly synthesize the *APOE* gene product within the central nervous system and have been recognized as the determinant contributing to the accelerated age-related cognitive decline in individuals. A correlation has been shown between *APOE* mutations and the occurrence of chronic pain in humans (Stuart et al., 2013; Gupta et al., 2009). ApoE is capable of promoting the efflux of intracellular cholesterol, which accumulates in microglial cells after the phagocytosis of dead cells and myelin debris, contributing to the inflammatory response (Tansley et al., 2022). The regulatory role of *APOC1* in the inflammatory process involves the augmentation of toll-like receptor 4 (TLR4)-dependent inflammation (Berbée et al., 2006; Curocichin et al., 2011), as well as the generation of pro-inflammatory cytokines and reactive oxygen species (ROS) (Tanga et al., 2005). The study determined that the lipid transport system may potentially undergo physiological alterations as a result of genetic factors.

The results of the present study indicate a prominent enrichment of 8 pleiotropic genes in brain tissue, suggesting that the central nervous system plays a significant role in the comorbid relationship between chronic pain and cognitive decline. Previous research has demonstrated significant hippocampal atrophy, a key brain region for memory, in individuals with chronic pain (Zhao et al., 2023; Xiao et al., 2023). Additionally, studies have shown a clear connection between cognitive impairment and brain imaging abnormalities in chronic pain sufferers. For instance, Baliki et al. observed disruptions in the dynamic function of the default mode network (DMN) in chronic pain patients, which may be associated with cognitive and behavioral impairments, such as decision-making deficits and attention problems (Baliki et al., 2008). Notably, the prefrontal cortex (PFC) has been implicated in both chronic pain and cognitive dysfunction, suggesting that this brain region may serve as a bridge linking these two conditions (Ong et al., 2019; Seminowicz and Moayedi, 2017). Functional enrichment analysis of these pleiotropic genes reveals their involvement in regulating neuron projection development and synapse organization. Given that chronic pain treatment has been shown to rely on proactive self-management (Miller et al., 2020; Hayward and Stynes, 2021), future research could investigate how such interventions affect neuronal structure and synaptic plasticity, particularly in key brain regions like the hippocampus and prefrontal cortex. Integrating genetic findings with brain imaging abnormalities could provide a more comprehensive understanding of the mechanisms linking chronic pain and cognitive dysfunction.

This study has several limitations. One notable limitation is the potential influence of environmental confounders, such as cross-trait assortative mating. While the LDSC method is designed to mitigate the impact of environmental factors, it cannot completely eliminate these biases. Such confounding effects may lead to an underestimation of genetic correlations. Future studies should incorporate complementary methods or conduct sensitivity analyses to address these potential biases and enhance the robustness of conclusions. Additionally due to the lack of GWAS specifically targeting Asian populations, this study utilized data derived from European populations. Although cognitive assessments may involve subjective bias, this limitation was minimized by employing rigorous and systematic evaluation methods, as well as leveraging a large sample size to ensure the reliability of the findings. Furthermore, considering prior epidemiological evidence indicating the significant impact of chronic multi-site pain on cognitive abilities, this study conducted genetic overlap analyses to investigate the relationship between multi-site pain GWAS and cognitive performance. Future research, contingent on data availability, could delve deeper into the influence of genetic factors on specific pain sites and their effects on cognitive function. At last, this study employed the COLOC method for colocalization analysis. However, it assumes a single causal variant per genomic region, which poses limitations in scenarios involving multiple causal variants. Future studies could adopt more advanced approaches, such as SuSiE, which models multiple causal variants using credible sets and posterior inclusion probabilities. These methods may improve the accuracy and robustness of colocalization analyses, providing more nuanced insights.

## 5 Conclusion

This study reveals a negative genetic correlation between chronic multisite pain and cognitive abilities. Additionally, shared genetic structures between chronic pain and cognitive functions were identified at the levels of SNPs, genes, and functional pathways. Building on these findings and related research, further investigation is needed to explore the potential of cognitive training in enhancing cognitive function in individuals with chronic pain and its effectiveness in mitigating cognitive decline associated with prolonged pain.

## Data Availability

The original contributions presented in the study are included in the article/Supplementary material, further inquiries can be directed to the corresponding author.

## Footnotes

^1^ https://github.com/zhenin/HDL

^2^ https://github.com/zhenin/HDL/wiki/Reference-panels

^3^ http://gusevlab.org/projects/fusion/
